# Does It Really Matter Where You Look When Walking on Stairs? Insights from a Dual-Task Study

**DOI:** 10.1371/journal.pone.0044722

**Published:** 2012-09-06

**Authors:** Veronica Miyasike-daSilva, William E. McIlroy

**Affiliations:** 1 Department of Kinesiology, University of Waterloo, Waterloo, Ontario, Canada; 2 Mobility Team, Toronto Rehabilitation Institute, Toronto, Ontario, Canada; 3 Heart and Stroke Foundation Centre for Stroke Recovery, Toronto, Ontario, Canada; California Pacific Medicial Center Research Institute, United States of America

## Abstract

Although the visual system is known to provide relevant information to guide stair locomotion, there is less understanding of the specific contributions of foveal and peripheral visual field information. The present study investigated the specific role of foveal vision during stair locomotion and ground-stairs transitions by using a dual-task paradigm to influence the ability to rely on foveal vision. Fifteen healthy adults (26.9±3.3 years; 8 females) ascended a 7-step staircase under four conditions: no secondary tasks (CONTROL); gaze fixation on a fixed target located at the end of the pathway (TARGET); visual reaction time task (VRT); and auditory reaction time task (ART). Gaze fixations towards stair features were significantly reduced in TARGET and VRT compared to CONTROL and ART. Despite the reduced fixations, participants were able to successfully ascend stairs and rarely used the handrail. Step time was increased during VRT compared to CONTROL in most stair steps. Navigating on the transition steps did not require more gaze fixations than the middle steps. However, reaction time tended to increase during locomotion on transitions suggesting additional executive demands during this phase. These findings suggest that foveal vision may not be an essential source of visual information regarding stair features to guide stair walking, despite the unique control challenges at transition phases as highlighted by phase-specific challenges in dual-tasking. Instead, the tendency to look at the steps in usual conditions likely provides a stable reference frame for extraction of visual information regarding step features from the entire visual field.

## Introduction

Many accidents during stair walking are attributed to perceptual errors and distractions [1] illustrating the importance of visual information during stair walking. Gaze behaviour studies indicate that people evenly look across the steps in a staircase, and that the gaze fixation point is maintained a few steps ahead in the path supporting the importance of foveal vision in continuously guiding immediate stepping [2], [3]. Additionally, fixating on the tread of the steps seems a gaze strategy that plays a dual role in providing visual input for appropriate foot placement and balance control [4]. Despite this potential role for foveal vision, there are times in everyday life when successful stair walking can be performed with the view of the steps unavailable (e.g., walking with boxes, laundry basket).

The prevalence of gaze behaviour documented in previous stair-related studies [2]–[4] may simply be the product of natural gaze tendencies in a familiar task and predictable environment rather than an index of reliance on foveal inputs. For example, the increased amount of time that people spend looking at the steps during ascent compared to descent [2] could be the result of the steps being naturally available in the visual field for longer time during stair ascent. Consequently, the overall gaze behaviour may overestimate the importance of foveal information during the control of stair locomotion.

The current study explored the role of foveal vision during stair locomotion by investigating the impact of diverting gaze in order to perform a concurrent visual task. Although this study was designed to explore the particular role of foveal vision on stair walking, dual-tasking could also have a confounding effect on the executive function [5]. In order to control for the influences of gaze direction and executive challenge, three levels of dual-task conditions were compared in this study: (1) visual reaction time (RT) task (gaze fixation and executive challenge), (2) stationary target fixation (gaze fixation but no executive challenge), and (3) auditory RT task (no gaze fixation and executive challenge). Overall, it was hypothesized that gaze directed towards the stairs would be less frequent when dual-task requires gaze fixations whether or not there was an executive challenges (i.e., target fixation comparable to visual RT task, and both different from auditory RT task).

In contrast, foveal vision and executive demands could play a crucial role while making the transition from level ground to stairs and vice-versa (transitions). Given that transitions are commonly associated with accidents [6]–[8], the present study also focused on the specific role of gaze and executive demands during locomotion on stair transitions. It was expected that, during dual-tasking, gaze fixations on the steps would be preserved, particularly in the phases preceding the transitions. Additionally, with increasing challenge in the dual-task context, it was expected that individuals would adopt more conservative movement strategies characterized by increased handrail use and slower walking speed. It was also anticipated a reduction in reaction time task performance (i.e., longer reaction time; lower accuracy) specifically during transition phases where the visual and executive demands are expected to be the greatest.

## Results

### Gaze Behaviour

**Figure 1:**
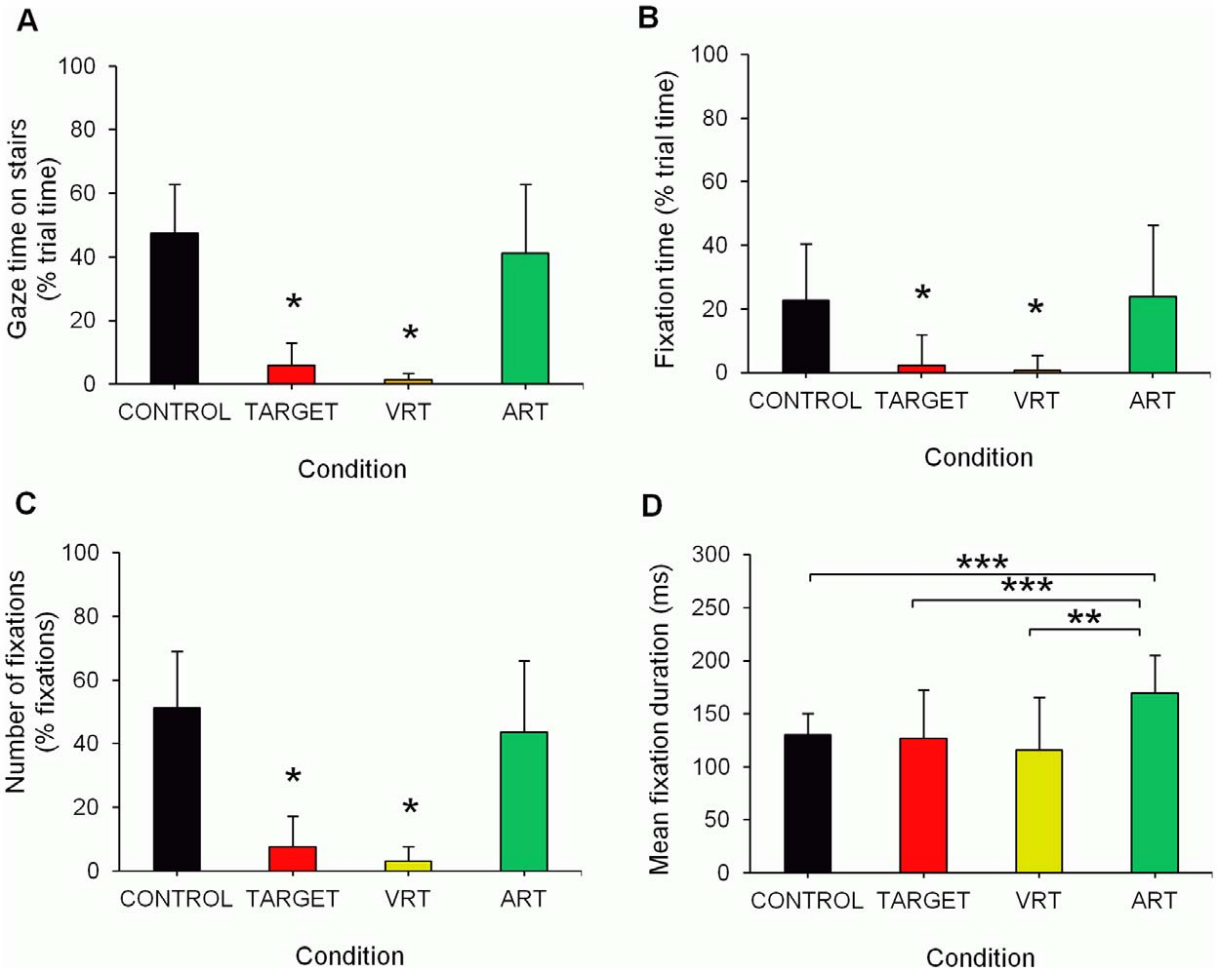
Effects of experimental conditions on gaze behavior. (a) total gaze time; (b) fixation time; (c) number of fixations; (d) fixation duration; ST = stair walking; TARGET = visual fixation target; ART = auditory reaction time; VRT = visual reaction time; *different from CONTROL and ART (p<0.0001); **p<0.01; ***p<0.05.

Total gaze time on the stairs (as percentage of trial time) was significantly influenced by task conditions (F(3,42) = 56.38, p<0.0001). Total gaze time was lower in TARGET and VRT compared to CONTROL and ART conditions (Figure 1a). Similarly, there were task related differences in total fixation time (F(3,42) = 42.92, p<0.0001), number of fixations (F(3,42) = 58.03, p<0.0001), and fixation duration (F(3, 35) = 5.33, p<0.005). Overall, TARGET and VRT showed reduced fixation time (Figure 1b) and number of fixations (Figure 1c) compared to ART and CONTROL. Conversely, fixations were significantly longer during ART compared to all other conditions (Figure 1d).

**Figure 2:**
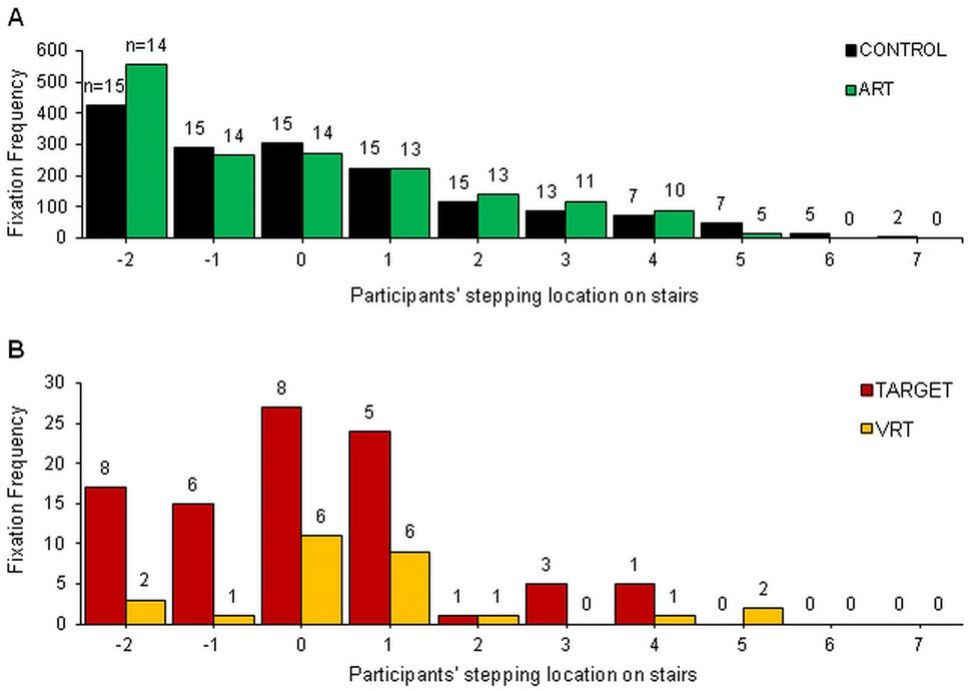
Frequency distribution of gaze fixations directed to the stairs relative to participants’ stepping location. Positive step numbers are the steps on the stairs. Frequency represents all fixations observed across all participants. Numbers at the top of the bars represent the number of participants contributing with fixations. Step “zero” represents the step ending with the last foot contact on the ground prior to the stairs. CONTROL and ART (A), and TARGET and VRT (B) were plotted in two difference graphs due to the large difference in scale.

Figure 2 shows the frequency of gaze fixations directed to specific steps on the stairs referenced to participants’ stepping location. For CONTROL and ART (Figure 2a), the greatest number of fixations occurred during the approach to the stairs and progressively reduced as participants continued ascent. When participants reached the last steps, they most often fixated at the end of the pathway following the stairs. For TARGET and VRT, fixation frequency was greater during the approach steps and initial transition (Figure 2b). However, the frequency of fixations and the number of subjects (numbers on the top of the bars) performing gaze fixations on the stairs were lower during TARGET and VRT compared to CONROL and ART.

### Locomotor Behaviour

**Figure 3:**
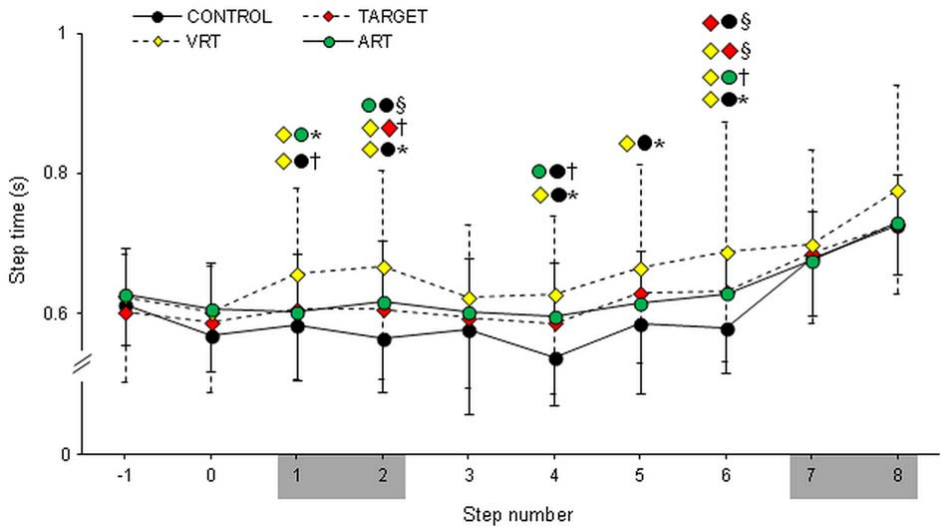
Step time across task conditions and step location. In the horizontal axis, positive step numbers are the steps on the stairs. Steps 1, 2, 7 and 8 represent the transition steps (shaded step numbers). Colored symbols indicate statistically different pairwise comparisons; §p<0.05;†p<0.01; *p<0.0001.

Task condition significantly influenced the time to walk on the stairs (F(3,42) = 4.94, p = 0.005; Figure 3). Total walk time on stairs was increased in VRT (6.64±1.16s) compared to CONTROL (6.01±0.69s; p = 0.002). ART (6.31±0.67s) and TARGET (6.26±0.93s) showed walk time values between CONTROL and VRT. Step time was different between tasks (F(3,42) = 4.82, p = 0.0056), the location of the step (F(9,126) = 32.56, p<0.0001) and there was a significant interaction between task condition and step location (F(27,378) = 2.95, 0<0.0001). Specifically, step time was longer in VRT compared to CONTROL, and on steps 1, 2, 4, 5, and 6.

Overall, only 3/15 participants contacted the handrail during the experiment in a few small number of trials, and these only occurred in TARGET (3 participants in 1, 2, and 5 trials, respectively) or VRT (1 participant in 9 trials) conditions. Interestingly, all the 3 participants used the handrail in their first trial of these task conditions. Inspection of the video recordings revealed that participants contacted the handrail when stepping on the first step, and slid their hands (left hand) across the length of the handrail until reaching the last step. Participants did not fixate on the handrails in any trial during this study even if they used the handrail.

### Reaction Time Performance

Overall, reaction time was significantly longer during dual-tasking compared to single-task in ART ((F(1,14) = 5076, p = 0.031; single-task: 319±27 ms; dual-task: 340±40 ms), but not in VRT (p = 0.097; single-task: 307±23 ms; dual-task: 319±24 ms). Accuracy was significantly reduced during dual-task compared to single-task in both ART (F(1,14) = 15.33, p = 0.002; single-task: 93.1±6.3%; dual-task: 87.0±5.5%), and VRT (F(1,14) = 25.7, p<0.001; single-task: 95.0±3.5%; dual-task: 87.3±6.0%).

**Figure 4:**
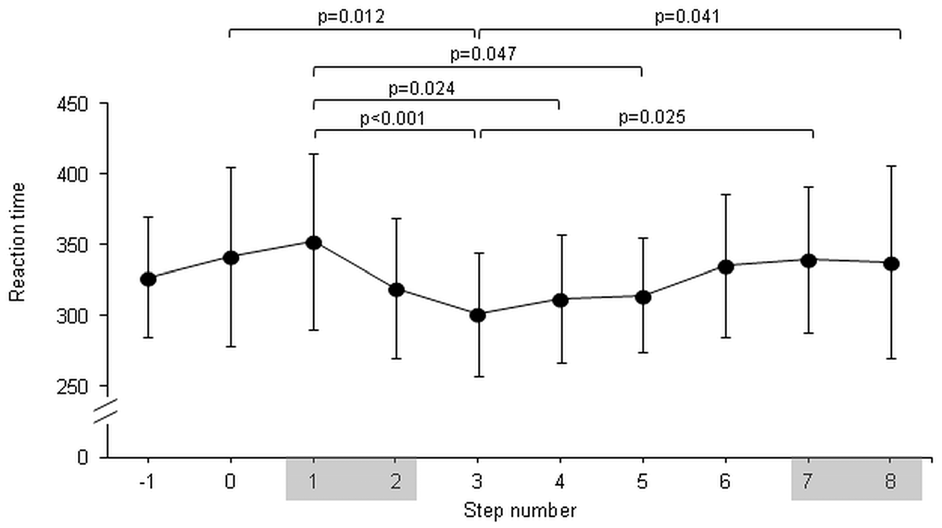
Reaction times referenced to location on the stairs. Horizontal axis defines the step number in which reaction time stimuli occurred. Positive numbers refer to steps on the stairs. Steps 1, 2, 7 and 8 represent the transition steps (shaded).

Reaction time was significantly different across stepping locations (F(9,126) = 3.94, p = 0.0002). The main effect for condition (p = 0.105) and the interaction conditions vs. stepping location (p = 0.056) were not statistically different. Reaction time was significantly longer in step 1 compared to steps 3, 4, and 5, and in step 3 compared to steps 0, 7 and 8 (Figure 4). For accuracy, there was no significant differences across stepping locations.

## Discussion

This study investigated the role of gaze fixations during stair walking. This study showed that gaze fixations on stair features drastically decreased when individuals performed a visual task while stair walking. Despite the shift in gaze, participants were still able to successfully ascent the stairs without transiently shifting gaze back to the steps even at transition phases. Handrail use was rare and there was modest change in walk speed associated with the diverted gaze. The most profound differences in behaviour were evident at transitions phases but only when the dual-tasking involved both executive and visual challenges. The increase in reaction time (auditory and visual dual-tasks) on the transition steps highlights the phase specific demands for executive function when an individual transitions on and off the stairs. In contrast, walking on the middle steps appeared immune to dual task and visual task demands.

Previous studies demonstrated that individuals spend a significant amount of time looking at the steps during stair walking suggesting that foveal fixations are required to guide locomotion [2], [3]. However, using a dual-task paradigm, the current study revealed that foveal vision may not be an essential requirement to extract visual information to control locomotion. Why do individuals direct their foveal gaze at the stairs if it is not essential? There are likely advantages in keeping foveal vision directed to the steps that are independent of foveal information. One possibility is that centering foveal vision to the stairs ensures that the entire visual field can be used to extract relevant information. It has been demonstrated, for instance, that peripheral visual information is sufficient to implement alternate foot placement, even when an obstacle suddenly appears in the travel path [9], as well as to provide information for support surfaces in the event of loss of balance [10]. It is possible, therefore, that peripheral vision was the primary source of visual information to guide stair walking specifically when vision was diverted. In this context, peripheral visual information likely provided online exproprioceptive information to fine tune limb trajectory on the steps, similarly to reports from obstacle avoidance studies [9], [11], [12].

While the findings from this study appear to diminish the potential importance of foveal vision, we did observe an increase in gaze fixations during the auditory dual-task condition. While this may have been linked to increased demands for foveal information during dual-tasking, most of the fixations were directed to the last steps in the staircase which coincides with a comfortable height for line of gaze. It remains unclear if these fixations served to provide a stable frame of reference to use optic flow and full visual field information to guide locomotion or simply reflected a reduction in gaze shifts when executive function was directed to non-visual tasks.

Overall, foveal fixation to an external target had a relatively small effect on walk time suggesting that locomotion can be sustained even when foveal vision is not used to monitor the stairs. Thus, foveal vision (including fixations) may not be the major source of visual information to guide stair locomotion and detect step edges as previously suggested [4]. Alternatively, the peripheral visual field may provide sufficient visual information to guide locomotion, as participants walked upstairs looking at the computer monitor, the view of the stairs was at least partially available in the lower peripheral field. The auditory task had a similar small effect on step time, which is in agreement with a previous study on obstacle avoidance that found that young adults kept gait parameters (gait velocity and stride time) constant while performing an auditory Stroop task [13]. This modest dual-task cost on walking speed and lack of influence on RT and accuracy, demonstrates that the current auditory task and stair walking did not pose a high collective demand for attention resources. However, when the secondary task included vision as well as executive requirements (VRT), participants walked slower. This could not be simply attributed to the fixation to a target since similar changes in walking speed were not observed in the gaze fixation task. So one possible explanation for this finding is that the load in executive function caused a narrowing in the attentional visual field. Previous studies demonstrated that the useful visual field reduces when individuals have their central visual field engaged in attentional tasks [14], [15], which could have been the case in the VRT condition leading to a reduction in gait speed. Therefore, the control of locomotion is most greatly affected when the concurrent dual task places demands on gaze control, reliance on visual inputs and executive function resources. While the present study revealed that foveal vision may not be as critical to stair walking, it did highlight phase-specific demands on executive function. The increase in reaction time in the transition steps suggest that transitions may impose additional executive demands compared to the middle (steady state) steps. The fact that reaction time was not increased in the middle steps could be associated with an overall reduction of executive/visual challenges in the steady state region reflecting a reliance on an internal prediction and working memory of stair dimension. In contrast, the executive demands of transitions phases may be linked to the adaptive control of stepping to accommodate for changes in foot placement (vertical and horizontal). Similar accommodation is also observed in gait parameters, such as foot clearance, which is reduced in the mid steps in comparison with the first step [16].

The weak support for the reliance on foveal vision for stair walking and handrail use does lead to the view that peripheral field information may play an important role during stair walking. This may be related in the lab and everyday life to the familiarity of the environmental characteristics. The stairs and handrails in this study were specifically selected to match standard stair design guidelines. As a result, reliance on an internal representation to predictively guide actions on stairs may account for the lack of any meaningful dual-task cost during the middle steady-state phases of stair walking and reduced reliance on foveal vision. One may certainly expect that in situations of environmental uncertainty, though not typical in everyday stair walking, the reliance on foveal or peripheral vision may vary.

### Conclusions

In a typical set of stairs, young adults are able to successfully control gait with minimal need for foveal fixations directed to stair features even in transition phases. It is suggested that visual information acquired through the whole visual field is able to provide visual inputs necessary for control of locomotion on stairs. In contrast, there is evidence for increased executive demands associated with the transitions to and from stairs in contrast to the steady state phase of stair walking. This may be linked to a varied reliance on the use of internal representation of the stair dimensions to guide stepping and grasping movements during stair walking. The relative contributions of visual inputs and the differences in control across different aspects of stair walking provide a foundation to explore age-related increases in fall-risk when stair walking.

## Materials and Methods

### Ethics Statement

This study was approved by the Office of Research Ethics at the University of Waterloo.

### Participants

Fifteen healthy adults (26.9±3.3 years; 8 females) provided written consent to participate in the study. Participants reported no medical condition affecting their balance or ability to traverse stairs and had normal vision or vision corrected to normal with contact lenses.

### Protocol

**Figure 5:**
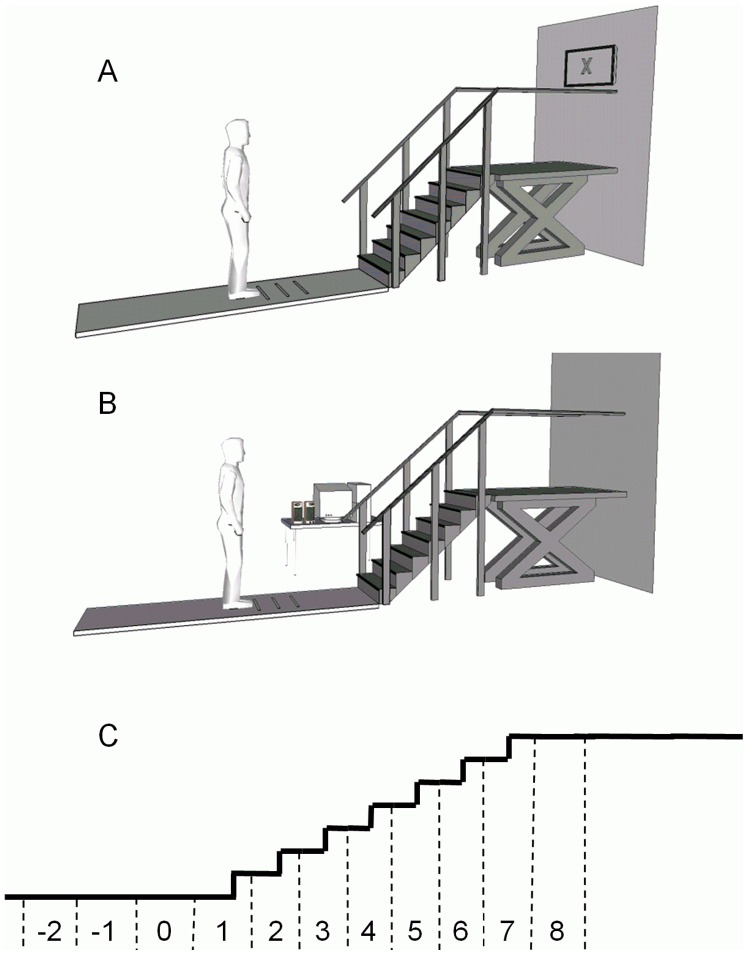
Schematics of the experimental setup. (a) stairs with monitor for presentation of visual stimuli for TARGET and VRT conditions. (b) In ART the monitor was occluded and computer speakers emitted the auditory stimuli. (c) Classification scheme for stepping location when ascending the stairs. Steps 0, −1, and −2 represent the steps in the approach. Steps 1 to 8 are the steps on the stairs. Steps 1 and 2 = first transition; Steps 7 and 8 = second transition.

Participants ascended a staircase with 7 steps (width: 96.5 cm; rise: 18 cm; tread: 25.5 cm) and handrails. A walkway was extended at the bottom step and a lift table at the same level of the top step provided an elevated walkway (Figure 5). Participants wore a safety harness attached to a retractable lanyard.

Stair walking was performed under 4 conditions (Figure 5a and b): 1) no secondary task (CONTROL); 2) visual target (TARGET): walking while fixating on the letter “X” presented on a monitor mounted on the wall at the end of the walkway; 3) visual reaction time (VRT): walking while performing a visual go/no go reaction time task; and 4) auditory reaction time (ART): walking while performing a auditory go/no go reaction time task. In VRT condition, the stimulus was either the letter “X” or “O” randomly presented on the monitor, while in ART, the stimulus was either a high or a low frequency tone randomly emitted by computer speakers. Participants were asked to click on a wireless mouse button every time they saw the “X” (VRT) and when they heard the high tone (ART). Each stimulus was presented for 100 ms at random time intervals between 750 and 1250 s. Proportion of occurrence was 3∶1 for both X/O stimuli (VRT) and high/low tones (ART).

Before each trial, a screen was held in front of the participant to prevent him/her from viewing the stairs, and the participant was asked to stand randomly at 1.5 m, 1.75 m or 2.0 m from the bottom step to prevent pre-planning of step lengths. Participants walked at their comfortable pace and carried the wireless mouse freely in their preferred hand in all four conditions. Participants, were instructed to perform the secondary task and stair walking concurrently, with no specific instruction on which task they should prioritize or where they should look.

The four task conditions (CONTROL, TARGET, ART, and VRT) were presented in blocks and the order of the blocks was randomized. For CONTROL and TARGET, participants performed 5 trials in each block. Within ART and VRT blocks, participants randomly performed 10 trials of dual-task (reaction time task + stair walking), and 5 trials of single-task (i.e., reaction time task only while standing still at beginning of the pathway for 10 seconds). VRT and ART blocks comprised of more trials than in CONTROL and TARGET blocks to allow sufficient number of stimulus-response events across the stairs for data analysis.

### Data Acquisition and Analysis

Eye movements were recorded using a head-mounted eye-tracker 5000 (ASL, USA) at 30 Hz. The eye-tracker was calibrated using the 9-point calibration method with 1° accuracy over the stair area. Footswitches (B&L Engineering, USA) placed inside of participant’s shoes provided foot contact times. A customized LabVIEW program (National Instruments, USA) recorded footswitch data, button press responses, and controlled the presentation of the visual and auditory stimuli on the monitor.

A frame-by-frame analysis of the gaze recordings identified gaze location and the mean gaze time was calculated for each step of the stairs. Gaze fixations were computed when gaze remained stationary for 67 ms or longer with maximal deviation of 1° of visual angle. The number of fixations (percentage of the total number of fixations), mean fixation duration, and total fixation time (percentage of the trial duration) were calculated for each step of the stairs.

Step time was calculated from foot contact to foot contact for the last three steps in the approach phase (−2, −1, and 0) and for each step on the stairs (1 to 8; Figure 5c). Steps 1 and 2 (bottom), and steps 7 and 8 (top) were defined as Transitions 1 and 2, respectively.

Reaction time and accuracy (% correct) were calculated for the auditory and visual reaction time tasks. Mean reaction time and accuracy were also calculated for each step location by taking the stimulus delivery time.

Gaze variables were analyzed by a one-way ANOVA with task condition as the factor. Frequency distribution of gaze fixations directed to the stairs was computed according to participants’ stepping location on the stairs in each condition. Step time was analyzed by a two-way ANOVA (task condition × step location). Reaction times and accuracy were analyzed by a one-way ANOVA to examine single-task/dual-task effects. Reaction time and accuracy were further analyzed by two-way ANOVA to evaluate the effect of task (ART, VRT) and step location. Tukey’s post-hoc analysis was performed to determine task or step location differences. Significance level was set at 0.05 for all analyses.
